# A169 THE ASSOCIATION OF FAECAL CALPROTECTIN MEASUREMENTS WITH ENDO-HISTOLOGICAL REMISSION IN ULCERATIVE COLITIS: A POOLED ANALYSIS.

**DOI:** 10.1093/jcag/gwae059.169

**Published:** 2025-02-10

**Authors:** L Dubé, P Lakatos, W Afif, A Bitton, G Wild, F Magro, A Patel, J Guardiola, T Bessissow

**Affiliations:** Internal Medicine, McGill University, Montreal, QC, Canada; Internal Medicine, McGill University, Montreal, QC, Canada; Internal Medicine, McGill University, Montreal, QC, Canada; Internal Medicine, McGill University, Montreal, QC, Canada; Internal Medicine, McGill University, Montreal, QC, Canada; Instituto de Farmacologia e Terapeutica, Porto, Portugal; Brooke Army Medical Center, Fort Sam Houston, TX; Hospital Universitari de Bellvitge, L’Hospitalet de Llobregat, Catalunya, Spain; Internal Medicine, McGill University, Montreal, QC, Canada

## Abstract

**Background:**

Endoscopic and histologic evaluation is standard practice to confirm the presence of active disease in ulcerative colitis (UC) before treatment. Fecal calprotectin (FCAL) levels show a positive correlation with endoscopic indices, relapse, and response to treatment. Meta-analyses on the diagnostic yield of FCAL for histologic remission are hampered by the different cut-offs used by existing studies.

**Aims:**

Our study aims to select an optimal FCAL threshold to discriminate between active disease or remission in UC based on Mayo endoscopic score (MES) and Geboes histologic score (GHS).

**Methods:**

We performed a pooled analysis of retrospective and prospective studies of patients with UC from 4 academic centres. Key inclusion criteria were adult UC patients undergoing colonoscopy, with recent FCAL measurements. Receiver operating characteristics curves and area under the curve (AUC) were computed to determine the optimal FCAL threshold for prediction of active UC based on specificity (SP) and sensitivity (SN). Active endoscopic disease was assessed using MES 0-1 vs 2-3 and MES 0 vs 1-3. Active histologic disease was assessed using GHS ≥ or < 2.0 and GHS ≥ or <3.1.

**Results:**

A total of 741 patients with UC were included in our analysis. For MES 0 vs 1-3, the AUC was 0.730 (95% CI; 0.686-0.775) with an optimal threshold of 145.2 µg/g (SP: 0.68, SN: 0.68). For MES 0-1 vs 2-3, an AUC of 0.806 (95% CI; 0.759-0.852) was computed with an optimal FCAL threshold of 215.3 µg/g (SP: 0.71, SN: 0.71). For GHS <2.0 vs GHS ≥2.0, the AUC was 0.656 (95% CI; 0.614-0.698) with an optimal threshold of 118.0 µg/g (SP: 0.60, SN: 0.60). For GHS <3.1 vs GHS ≥3.1, the AUC was 0.752 (95% CI; 0.712-0.792) with an optimal threshold of 164.7 µg/g (SP: 0.70, SN: 0.70).

**Conclusions:**

Our analysis suggests that FCAL has the potential to be used in the clinical setting to determine the likelihood of concurrent endoscopic and/or histological disease activity. We plan on testing and validating our thresholds in an independent dataset to assess the clinical relevance of FCAL in predicting UC remission and in guiding physicians in pursuing further tests such as endoscopy and biopsy.

**Funding Agencies:**

None

